# Bridging evidence-practice gaps: improving use of medicines in elderly Australian veterans

**DOI:** 10.1186/1472-6963-13-514

**Published:** 2013-12-12

**Authors:** Elizabeth E Roughead, Lisa M Kalisch Ellett, Emmae N Ramsay, Nicole L Pratt, John D Barratt, Vanessa T LeBlanc, Philip Ryan, Robert Peck, Graeme Killer, Andrew L Gilbert

**Affiliations:** 1 Quality Use of Medicines and Pharmacy Research Centre, School of Pharmacy and Medical Sciences, Sansom Institute, University of South Australia, Adelaide, Australia; 2 Data Management and Analysis Centre, Discipline of Public Health, University of Adelaide, Adelaide, Australia; 3 Department of Veterans’ Affairs, Canberra, Australia

**Keywords:** Health promotion, Quality improvement, Quality use of medicines, Translational research, Clinical audit, Evidence-based practice

## Abstract

**Background:**

The Australian Government Department of Veterans’ Affairs (DVA) funds an ongoing health promotion based program to improve use of medicines and related health services, which implements interventions that include audit and feedback in the form of patient-specific feedback generated from administrative claims records. We aimed to determine changes in medicine use as a result of the program.

**Methods:**

The program provides targeted patient-specific feedback to medical practitioners. The feedback is supported with educational material developed by a clinical panel, subject to peer review and overseen by a national editorial committee. Veterans who meet target criteria also receive educational brochures. The program is supported by a national call centre and ongoing national consultation. Segmented regression analyses (interrupted time series) were undertaken to assess changes in medication use in targeted veterans pre and post each intervention.

**Results:**

12 interventions were included; three to increase medicine use, seven which aimed to reduce use, and two which had combination of messages to change use. All programs that aimed to increase medicine use were effective, with relative effect sizes at the time of the intervention ranging from 1% to 8%. Mixed results were seen with programs aiming to reduce inappropriate medicine use. Highly specific programs were effective, with relative effect sizes at the time of the intervention of 10% decline in use of NSAIDs in high risk groups and 14% decline in use of antipsychotics in dementia. Interventions targeting combinations of medicines, including medicine interactions and potentially inappropriate medicines in the elderly did not change practice significantly. Interventions with combinations of messages targeting multiple components of practice had an impact on one component, but not all components targeted.

**Conclusions:**

The Veterans’ MATES program showed positive practice change over time, with interventions increasing use of appropriate medicines where under-use was evident and reduced use of inappropriate medicines when single medicines were targeted. Combinations of messages were less effective, suggesting specific messages focusing on single medicines are required to maximise effect. The program provides a model that could be replicated in other settings.

## Background

The difficulty in translating research findings to the practice setting has been well described [1,2], particularly in the area of medicines use. Under-prescribing of effective medicines is common, as is use of too much medicine, use of the wrong drug, and use of an inappropriate medicine regimen [3,4]. Bridging the evidence-practice gap in the area of medicines-related health care is critical because medicines are the most commonly used health care intervention [5], and there is a significant gap between existing evidence and practice in relation to medicine use [6]. Implementation research provides evidence to guide the design of quality improvement programs, which can promote the translation of research findings to practice and improve the use of medicines, patient care, and health outcomes [1].

Audit and feedback are often promoted to improve use of medicines; however, systematic reviews of interventions to improve uptake of evidence in practice have found mixed success [1,7]. A systematic review of randomized controlled trials of audit and feedback found that some interventions were associated with a large increase in adherence to practice guidelines, while others had a negative effect [7]; the median relative percentage practice improvement was 8 percent, with the adjusted risk ratio varying from 0.7 to 18 across studies [7]. The duration of effect following intervention also varied, with improvements in practice seen in some studies at up to six months follow-up, while in other studies there was no difference between intervention and control groups at three weeks post intervention [7]. Some studies found no difference between intervention and control groups at any point of time during follow-up [7]. Audit and feedback interventions were more likely to have an effect when baseline adherence to the targeted treatment guideline was low, however, the effect was still modest [7]. This may be because many implementation studies do not consider communication, behavioral, and health promotion theories in their design. One review of guideline implementation studies found that less than 10 percent of studies identified the theoretical rationale underpinning the intervention [1]. While results vary, collectively, the evidence suggests audit and feedback is effective. From an implementation science perspective, the next research question becomes how to implement audit and feedback routinely as part of ongoing routine health-care improvement.

In this paper we report the results of an ongoing health promotion–based quality improvement program that uses audit and feedback in the form of patient specific feedback generated from administrative claims data to improve use of medicines in the elderly Australian war veteran population. Since 2004, the Australian Government Department of Veterans’ Affairs (DVA) has funded a quality improvement program, the Veterans’ Medicines Advice and Therapeutics Education Services (Veterans’ MATES) program [8,9], to bridge the evidence-practice gap in the provision of health care to Australian war veterans. The overall aim of this paper is to determine changes in medicine use as a result of the program.

## Methods

**Figure 1:**
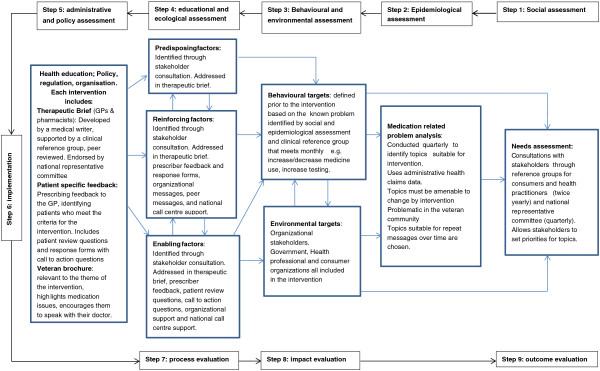
Components of veterans’ MATES interventions, linked to steps in the precede-proceed model.

**Figure 2:**
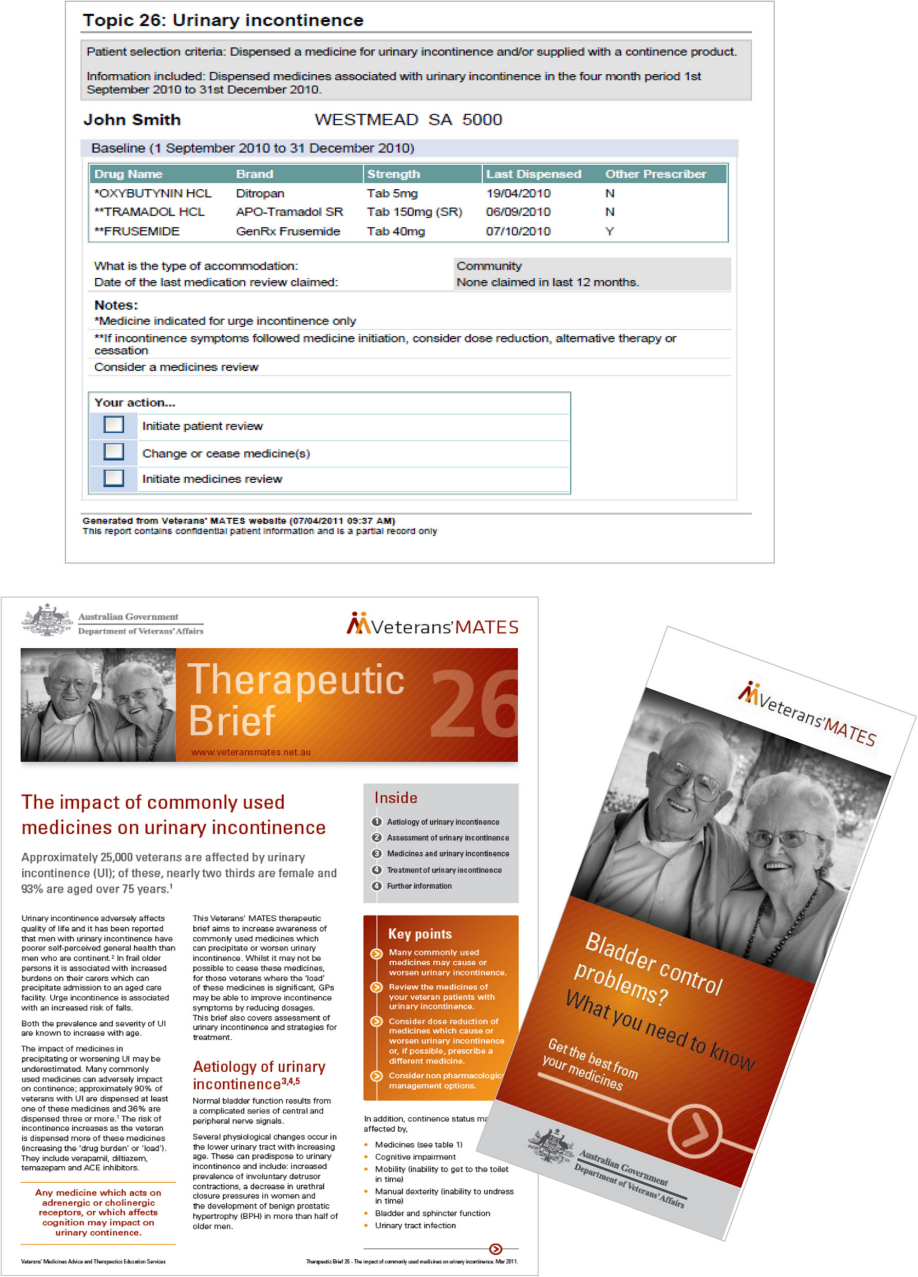
Intervention materials provided for the veterans’ MATES program.

The Veterans’ MATES program aims to improve medication use and health outcomes for all persons in the veteran community by delivering interventions to general practitioners (GPs), pharmacists, and veterans. Social cognitive theory [10,11], the transtheoretical model [12,13], and the health promotion model Precede-Proceed [14] were used as the theoretical frameworks that underpinned the program and were used to predict learning and behaviour change (Figure 1). Key features of the implementation design included the ability to provide routine periodic interventions with ongoing evaluation suitable for participation by all practitioners (Figure 1). The main intervention was patient-specific feedback that identified for GPs their patients with potential medication-related problems. The feedback included a list of the patient’s relevant medicines, contained notes identifying the potential problems, and included tick boxes for GPs to indicate the actions they would take in response to the information, including the need for a review of the patient’s therapy (Figure 2). Supportive educational material was also provided that included advice to assist the GP in resolving the potential medication-related problem. In addition, the veterans identified in the GP mailing were mailed an educational brochure highlighting potential medication issues related to the topic and encouraging the veterans to speak with their doctor. The same educational material was also provided to all pharmacies and accredited pharmacists to enable pharmacists to support the practice change (Figure 2). The program was implemented four times per year. Mailings were delivered only to those GPs and veterans who met the criteria for each program and to all pharmacies and accredited pharmacists. Between November 2004 and September 2008, 16 educational interventions were distributed. Twelve of these interventions focused on changing medication use and are the subject this paper. The aims of the twelve interventions are listed in Table one. Four other interventions focused on health service use, including home medicines review and dose administration aids, and are not reported here. For the interventions, material was mailed first to GPs and pharmacists, with the educational material to veterans provided one month later to enable GPs to read the material prior to the veteran visit.

In keeping with health promotion principles involving needs assessments and identifying barriers and enablers to change, the program began with nationwide consultation to stakeholder groups, including health professional and consumer organizations (Figure 1). The program also established practitioner and consumer reference groups which meet twice a year to provide advice on topics, interventions, and practitioner and consumer needs. The educational material is developed by a medical writer, supported by a clinical reference group that meets monthly. Prior to publication, the material is peer reviewed and also reviewed by a national representative editorial committee to ensure both the quality of the material and stakeholder support for the material and intervention. The program is further supported by a national call centre staffed by clinical pharmacists who provide educational support, clarify issues, ensure problems are quickly identified, and accept complaints. National consultation with stakeholder organizations and advice from veteran and practitioner reference groups is ongoing (Figure 1).

Each educational intervention is based on a specific medicine or health-related topic. To identify topics, the prevalence of medication-related problems is determined using DVA administrative health claims data (Figure 1). In 2004 when the program commenced, there were approximately 320,000 Australian war veterans, war widows, or widowers. Their median age was 80 years; over 40 percent regularly used two or more medicines, and 13 percent regularly used five or more medicines. With veterans aged 70 years and over dispensed an average of 45 prescriptions per year, the population is at high risk of medication-related problems because of their high medication use. DVA health claims data contain details of all DVA subsidized prescription medicines, medical and allied health services, and hospitalizations for veterans. Veterans are entitled to receive all medicines subsidized on Australia’s national pharmaceutical benefits scheme and entitled to receive additional medicines that are subsidized under the Repatriation Pharmaceutical Benefits Scheme. DVA maintains a client file, which includes data on gender, date of birth, date of death, and family status. Medicines are coded in the data set according to the World Health Organization (WHO) anatomical and therapeutic chemical (ATC) classification [15] and the Schedule of Pharmaceutical Benefits item codes [16]. Hospitalizations are coded according to the WHO International Classification of Diseases [17].

The medication-related problem analyses were generally based within the therapeutic areas outlined in the Australian government National Health Priority Areas (asthma, cardiovascular health, diabetes, mental health, and arthritis and musculoskeletal conditions) [18]. Criteria for each topic were that it was problematic in the veteran community, specific to medication or health service management, amenable to change by the interventions employed by the Veterans’ MATES program, and able to be evaluated using DVA administrative data sets. In addition, the topics chosen were suitable for repeat messages over time, to increase the persuasiveness of the messages [19]. In developing the intervention, strategies consistent with behavioural theories were established. Every educational intervention included strategies to raise awareness, improve knowledge, and encourage trial of appropriate behaviours. Each intervention included objectives to provide useful information to participants; to increase GPs’ knowledge of the veterans they treat who meet the target criteria for the intervention; and to change medication or health service use in line with the messages of the educational intervention. GPs and pharmacists could claim professional development points for participating. This provided an incentive for GPs and pharmacists as professional development reports are required for maintaining practicing certificates. Table 1 provides the final list of interventions and target groups.

**Table 1:** Educational intervention topics and target audience

**Intervention number and topic**	**GPs (n)**	**Veterans (n)**	**Pharmacists (n)**	**Aim of educational intervention**	**Target criteria for veterans mailed to:**
**Interventions to increase use of medicines**					
2: Beta-blockers, take the next step for heart failure	6954	12047	N/A	To increase beta-blocker use in veterans with heart failure	Veterans dispensed medicines indicative of heart failure who were not dispensed a heart failure specific beta blocker
3: Diabetes triple check	8573	16612	5459	To increase use of adjunct cardiovascular medicines in veterans with diabetes	Veterans dispensed medicines indicative of diabetes (insulin and/or oral hypoglycaemics)
15: Osteoporosis	16876	83110	7967	To increase uptake rates of bone density tests, and osteoporosis treatments	Women aged 70–79, men aged 80–85 and those over 50 years admitted to hospital with a fracture from a same level fall
**Interventions to reduce use of medicines**					
4: Clinical risk management: NSAIDs	11242	9885	5447	To reduce NSAID use in veterans with heart failure and diabetes	Veterans with dispensed medicines indicative of diabetes and/or heart failure
5: Antidepressants: three steps towards safer use	12482	42196	5447	To reduce potentially avoidable antidepressant interactions and duplicate therapy	Veterans dispensed antidepressants
6: Inhaled respiratory medicines: optimising use	10720	28670	5447	To decrease use of multiple devices	Veterans dispensed inhaled respiratory medicines
7: PPIs in GORD: Reduce the dose – keep the benefits	13684	62460	5447	To encourage use of lower-strength PPIs for maintenance therapy	Veterans dispensed proton pump inhibitors
8: Reducing adverse drug events for your veteran patients	11050	32484	7074	To reduce use of potentially inappropriate medicines in the elderly (aged over 70 years)	Veterans 70 years of age or over, dispensed medicines that should be used with caution in the elderly (according to Beers [33] and McLeod [34] criteria)
12: Antipsychotics in dementia	3884	6690*	8089	To reduce antipsychotic use for behavioural and psychological symptoms of dementia (in those aged over 65 years).	Veterans aged >65 years dispensed oral antipsychotic medicines.
14: COPD	8785	18096	7880	To reduce nebuliser use and reduce multiple device use	Veterans dispensed tiotropium or ipratropium
**Interventions with a combination of messages**					
10: Constipation: a quality of life issue for veteran patients	9825	29231	7327	To improve use of medicines for constipation; specifically, to increase the use of osmotic and bulk laxatives and reduce the use of contact laxatives.	Veterans dispensed laxatives
13: Clopidogrel	8279	16867	7970	To increase use of clopidogrel with aspirin and reduce concurrent use of clopidogrel with NSAIDs	Veterans dispensed clopidogrel

*Material was not mailed to veterans. For this topic, materials were provided to the GPs treating these 6990 patients to pass on to the patient if appropriate.

NSAID, non-steroidal anti-inflammatory drug; PPI, proton pump inhibitor; GORD, gastro-oesophageal reflux disease; COPD, chronic obstructive pulmonary disease.

### Evaluation

Stakeholder satisfaction was evaluated for all interventions using a one-page reply paid response form that was mailed with the educational materials. The evaluation forms were designed to minimize additional workloads for busy health professionals, thus, the forms were limited to no more than nine questions (one page) and all answers required a “tick-box” response only. Evaluation forms were created for every intervention with questions relevant to the targeted topic. Specific forms were created for GPs, pharmacists, and veterans. All targeted participants were mailed the evaluation forms. No follow-up reminders were sent. The response forms included questions on the usefulness of the educational material, as well as likely actions to be taken as a result of the program. Three months after each intervention, responses were collated. Individuals could only respond once for each intervention but could respond to multiple interventions if they had been targeted in more than one intervention.

### Statistical analyses

Segmented regression analysis was used to estimate the effect of each intervention on medicine utilisation taking into account the baseline trend prior to the intervention [20]. A log-binomial generalised estimating equation was used with an ar (1) error structure to account for correlation between months, clustered by patient. The models included a constant term, a term for baseline trend prior to the intervention, an indicator term to estimate the change in level at the time of the intervention and a post-intervention trend term to determine the sustainability of the intervention over time. We calculated the number of people estimated to have changed behaviour as a result of each intervention based on the number of veterans targeted at the time of the intervention, the level change at the time of the intervention plus the monthly trend change at the time after the intervention compared to the pre-intervention trend over the 24 month period. The time periods used for the analysis were as follows: the pre intervention period was the 24 months prior to the month of mail out for each intervention, the intervention period was the four months immediately following the month of mail out, and the post-intervention period was the subsequent 20 months (i.e. month 5 to 24 post mail-out). All analyses were undertaken using SAS for windows, V9.1.3 SP4 (SAS Institute, Cary, North Carolina, USA).

### Ethics statement

Ethics approval for the study was obtained from the Department of Veterans’ Affairs Human Research Ethics Committee (reference number: E004/016) and the University of South Australia Human Research Ethics Committee (reference number: P203/04).

## Results

The twelve interventions implemented had an average target group size for each intervention of 33,000 veterans, 10,000 doctors, and 8500 pharmacies/accredited pharmacists. The target group size and aim of each individual intervention is listed in Table 1.

**Figure 3:**
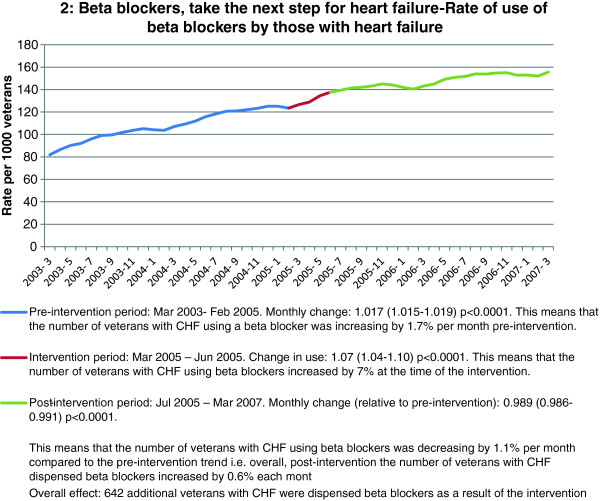
Example of an intervention aiming to increase medicine use.

Of the three interventions that aimed to increase medicine use, all achieved statistically significant increases at the time of the intervention. However, all post-intervention trends, while remaining positive, were lower than the pre-intervention trend (Table 2 and Figure 3). Estimated numbers of patients with sustained behaviour change over the subsequent 24 months as a result of the intervention ranged from 642 to 3234 per intervention.

**Table 2:** Impact of interventions to increase medicine use

**Intervention number and topic (number of veterans targeted)**	**Targeted therapy**	**Proportion of veterans on targeted therapy at baseline (%)**	**24 month pre-intervention trend**	**Intervention effect (%)**	**20 month post intervention trend**	**Estimated number with changed behaviour from intervention; sustained over two years post intervention**
2: Beta-blockers, take the next step for heart failure (n = 12047)	Increase beta-blocker use	8.7%	Trend increasing 1.7% per month	7% (p < 0.0001)	Trend still increasing but reduced to 0.6% per month (p < 0.0001)	642
3: Diabetes triple check (n = 16612)	Increase ACE inhibitor use	61.3%	Trend increasing 0.3% per month	1.3% (p < 0.0001)	Trend still increasing but reduced to 0.1% per month (p < 0.0001)	543
	Increase lipid lowering therapy use	47.9%	Trend increasing 0.6% per month	1.9% (p < 0.0001)	Trend still increasing but reduced to 0.5% per month (p = 0.015)	1573
	Increase antiplatelet use	41.9%	Trend increasing 0.4% per month	3% (p < 0.0001)	Trend still increasing but reduced to 0.1% per month (p < 0. 0.0001)	543
15: Osteoporosis (n = 83110)	Increase use in women	9.1%	Trend increasing 1.1% per month	2.8% (p < 0.004)	Trend still increasing but reduced to 0.4% per month (p < 0.0001)	712
	Increase use in men	4.7%	Trend increasing 1.5% per month	8.3% (p < 0.0001)	Trend still increasing but reduced to 0.2% per month (p < 0.0001)	2522

ACE inhibitor, angiotensin converting enzyme inhibitor.

**Figure 4:**
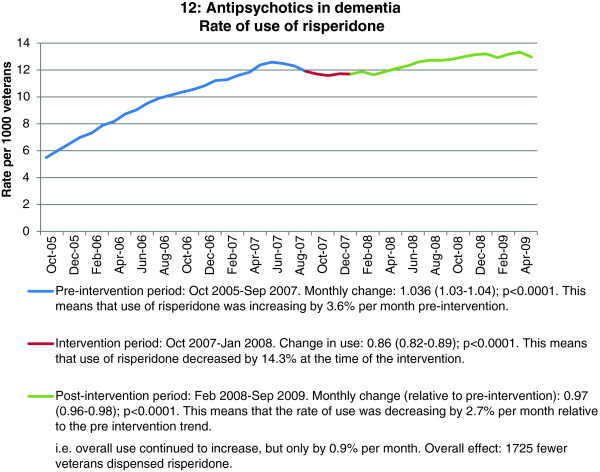
Example of an intervention aiming to reduce medicine use.

Of the seven interventions that aimed to reduce use, the three that targeted one specific medicine to be reduced, NSAID use in those with heart failure or diabetes, high dose proton pump inhibitor (PPI) use, and antipsychotic use in those aged over 65, all achieved significant level changes at the time of the intervention, ranging from 10% to 14% (Table 3 and Figure 4). For proton pump inhibitors and antipsychotics, the impact was increased over the 20 month follow-up period as evidenced by the statistically significant further reduction in the trends post intervention compared to pre-intervention trends (Table 3). The estimated number of patients with sustained behaviour change over 24 months ranged from 780 to 1725 for the three interventions. By comparison, the four interventions to reduce use that had multiple messages, medicine interactions (antidepressant interactions), potentially inappropriate medicines in those aged over 70 and respiratory device use did not show significant effects. Pre-intervention trends, which in most cases were falling, continued to fall. Duplicate antidepressant use was the exception, where usage increased despite messages that duplicate use was not appropriate. The two interventions that targeted multiple respiratory device use for those on four or more different devices only reduced post intervention, but not at the time of the intervention (Table 3).

**Table 3:** Impact of interventions to reduce use

**Intervention number and topic (number of veterans targeted)**	**Targeted therapy**	**Proportion of veterans on targeted therapy at baseline (%)**	**24 month pre-intervention trend**	**Intervention effect (%)**	**20 month post intervention trend**	**Estimated number with changed behaviour as a result of intervention; sustained over two years post intervention**
4: Clinical risk management: NSAIDs (n = 9885)	Reduce NSAID use in those with heart failure	15.6%	Trend decreasing − 0.8% per month	−11.4% (p < 0.0001)	Trend still decreasing (non-significant to prior) − 0.7% per month (p = 0.87)	1163 (680 heart failure patients and 483 diabetes patients)
	Reduce NSAID use in those with diabetes	17.9%	Trend decreasing − 1.2% per month	−10.2% (p < 0.0001)	Trend still decreasing but reduced to − 0.3% per month p = 0.006	
5: Antidepressants: three steps towards safer use (n = 42196)	Reduce antidepressant duplication	3.5%	Trend increasing 0.2% per month	0.6% p = 0.72	Trend increasing at a rate of 0.5% per month p = 0.05	No effect
	Reduce antidepressants with tramadol	5.8%	Trend decreasing − 0.6% per month	−1.0% p = 0.64	Trend still decreasing (non-significant to prior) − 0.8% per month p = 0.23	
6: Inhaled respiratory medicines: optimising use (n = 28670)	Reduce multiple device use (3 different devices)	9.5%	Trend increasing 0.2% per month	−0.11% p = 0.32	Trend now decreasing (non-significant to prior) − 0.2% per month p = 0.23	100 patients no longer on 4 or more different devices
	Reduce multiple device use (4 or more different devices)	2%	Trend increasing 0.1% per month	2% p = 0.60	Trend now decreasing − 0.6% per month p = 0.01	
7: PPIs in GORD: Reduce the dose – keep the benefits (n = 62460)	Reduce use of high dose proton pump inhibitors (measured as increase in low-dose use)	2.5%	Trend of low dose increasing 0.6% per month	14.5% (p < 0.0001) Increase in low dose use	Trend of low dose increasing at greater rate 0.9% per month (p = 0.007)	780
8: Reducing adverse drug events for your veteran patients (n = 32484)	Reduce use of potentially inappropriate medicines	14.7%	Trend decreasing − 0.2% per month	−0.3% p = 0.12	Trend still decreasing (non-significant to prior) − 0.2% per month p = 0.63	No effect
12: Antipsychotics in dementia (n = 6690)	Reduce antipsychotic use	0.54%	Trend increasing 3.6% per month	−14.3% (p < 0.0001)	Trend increasing but at a reduced rate 0.8% per month (p < 0.0001)	1725
14: COPD (n = 18096)	Reduce multiple device use (4 or more different devices)	2%	Trend increasing 0.1% per month	2% p = 0.68	Trend now decreasing − 1.1% per month p <0.0001	105 patients no longer on 4 or more different devices
	Reduce use of nebules	15.2%	Trend decreasing − 0.6% per month	2% p = 0.07	Trend decreasing (non-significant to prior) − 0.8% per month p = 0.2	

NSAID, non-steroidal anti-inflammatory drug; PPI, proton pump inhibitor; GORD, gastro-oesophageal reflux disease; COPD, chronic obstructive pulmonary disease.

Table 4 demonstrates the results of two interventions that had combined messages to increase one aspect of medicine use and decrease another. Significant changes were seen in keeping with intervention messages for clopidogrel at the time of the intervention with increased aspirin use and reduced NSAID use; subsequent trends remained unchanged. By contrast, laxative use did not change at the time of the intervention, but subsequent trends were statistically significant in keeping with intervention messages.

**Table 4:** Interventions with combination messages (to reduce and increase medicines use)

**Intervention number and topic (number of veterans targeted)**	**Targeted therapy**	**Proportion of veterans on targeted therapy at baseline (%)**	**24 month pre-intervention trend**	**Intervention effect (%)**	**20 month post intervention trend**	**Estimated number with changed behaviour as a result of intervention; sustained over two years post intervention**
10: Constipation: a quality of life issue for veteran patients (n = 29231)	Increase use of osmotic laxatives	1.5%	Trend increasing 0.7% per month	1.8% (p = 0.11)	Trend increasing at greater rate 1.2% per month (p = 0.002)	2047 (825 additional people on osmotic laxatives, 410 additional people on contact laxatives and 690 less people on bulk laxatives)
	Decrease use of contact laxatives	2.5%	Trend increasing 0.2% per month	3.0% (p = 0.0007)	Trend still increasing (non-significant to prior) 0.2% per month (p = 0.87)	
	Increase use of bulk laxatives	1.9%	Trend decreasing − 0.7% per month	0.8% (p = 0.34)	Trend now decreasing at greater rate − 0.9% per month (p = 0.04)	
13: Clopidogrel (n = 16867)	Increase use of clopidogrel with aspirin	1.5%	Trend increasing 0.8% per month	3.0% (p = 0.018)	Trend still increasing (non-significant to prior) 0.7% per month (p = 0.4)	1114 (825 now on aspirin with clopidogrel, 289 no longer on NSAIDs with clopidogrel)
	Reduce concurrent use of clopidogrel with NSAIDs	1%	Trend decreasing − 0.3% per month	−5.3% (p = 0.002)	Trend still increasing (non-significant to prior) 0.1% per month (p = 0.3)	

The stakeholder evaluations for each intervention had a median response rate of 8% (interquartile range 7%-10%) for GPs, 9% (7%-9%) for pharmacists and 26% (24%-32%) for veterans. On average, 80% of GPs and 93% of pharmacists who completed the stakeholder evaluation found the interventions useful or very useful (Table 5). On average, 80% of veterans who completed the stakeholder evaluation found the information provided helpful or very helpful (Table 5).

**Table 5:** Stakeholder evaluation: stakeholders who found the information “useful or very useful” (GPs and pharmacists) or “helpful or very helpful” (veterans)

**Intervention number and topic**	**GP feedback: useful or very useful**^**#**^	**Pharmacist feedback: useful or very useful**^**#**^	**Veteran feedback: helpful or very helpful**^**#**^
2: Beta-blockers, take the next step for heart failure	69%	N/A*	81%
3: Diabetes triple check	71%	92%	82%
4: Clinical risk management: NSAIDs	73%	95%	80%
5: Antidepressants: three steps towards safer use	81%	92%	81%
6: Inhaled respiratory medicines: optimising use	80%	91%	86%
7: PPIs in GORD: Reduce the dose – keep the benefits	81%	95%	72%
8: Reducing adverse drug events for your veteran patients	84%	95%	86%
10: Constipation: a quality of life issue for veteran patients	84%	92%	72%
12: Antipsychotics in dementia	85%	93%	N/A*
13: Clopidogrel	86%	98%	87%
14: COPD	79%	90%	77%
15: Osteoporosis	84%	92%	77%
Average	80%	93%	80%

^#^Percent of GPs, veterans or pharmacists who provided feedback.

*No feedback was sought from pharmacists for intervention 2 or from veterans for intervention 12.

## Discussion

In our study, all educational interventions aiming to increase medicine use were effective, with relative effect sizes ranging from 1% to 8% at the time of the intervention. However, all of the interventions also had a statistically significant effect on the post-intervention trend which was reduced compared to the pre-intervention trend. The reduced trend observed after the intervention could be due to the reduced naïve patient pool which results as a direct consequence of the immediate impact of the intervention. It also suggests that the effect of interventions to increase use of medicines may not be sustained and would require repeat messages over time to be sustained.

Educational interventions aiming to reduce unnecessary use of medicines had mixed results. Those with very specific messages (reduce use of non-steroidal anti-inflammatory agents in high risk patients; reduce use of high dose proton pump inhibitors; and reduce use of risperidone for the behavioural and psychological symptoms of dementia) were all effective, with relative effect sizes up to 14%. Educational interventions with more generic adverse event messages (avoid antidepressant interactions, reduce potentially inappropriate medicine use in the elderly) had no measurable effect on practice. Interventions less clearly linked to patient harm or poor health outcomes and with combinations of messages, such as those to reduce use of multiple inhaler devices, and improve the use of medicines for constipation were associated with practice change in some but not all aspects of care and were often found to be statistically significant in the post-intervention trend only, not at the time of the intervention. It is likely that the combination of messages meant these interventions were less effective. The evaluation of these interventions may also have been confounded by patient practice, where these medicines may be used “as required” and thus change in dispensing quantities may be delayed, hence the positive effect in the post-intervention trend only.

The relative effect of our results, which ranged between 1% and 14%, is consistent with the median relative effect reported in the Cochrane review of audit and feedback of 8%^7^. Our results suggest well implemented, behaviourally grounded, ongoing programs do achieve similar results to randomised control results.

The Veterans’ MATES program is a national program, targeting all general practitioners in Australia. Thus, the program requires sustained engagement of participants to maintain its success. The outcomes reported here demonstrate a bridging of existing evidence-practice gaps in diverse therapeutic areas. The behavioural intervention, utilising patient-specific prescriber feedback, is based on evidence from randomised controlled trials [7]. The implementation and evaluation frameworks were based on behavioural theories and have enabled implementation of evidence in practice. The significance of this work lies in its ability to bridge the research-practice gap with an implementation and evaluation framework that enables sustained engagement and demonstration of effectiveness.

The changes in medication use reported in this paper are associated with improvements in health outcomes. We have previously shown that PPI use in the Australian veteran population was associated with increased use of antibiotics (RR 1.23, 95% CI 1.21–1.24) and increased risk of hospitalisation for pneumonia (RR 1.16, 95% CI 1.11–1.22) [21]. We have demonstrated increased risk of hospitalisation associated with use of NSAIDs amongst veterans with diabetes (IRR 1.31, 95% CI 1.08 – 1.60) and veterans dispensed medicines indicative of heart failure (IRR 1.34, 95% CI 1.13 – 1.58) [22]. We have also demonstrated increased risk of death, stroke, hip fracture and pneumonia with antipsychotic use in the elderly veteran population [23-25]. We also demonstrated that beta-blocker use was associated with less hospitalisations for heart failure in elderly veterans [26]. The Veterans’ MATES program was associated with declines in use of proton pump inhibitors, reducing NSAID use, reducing antipsychotic use and increasing beta-blocker use in those with heart failure, all suggesting health outcomes for veterans have improved.

The implementation of the Veterans’ MATES program includes all the elements identified within the National Institute for Clinical Excellence Principles of Best Practice for Clinical Audit [27] and is consistent with the behavioural theories, particularly the Precede-Proceed Model of Health Promotion [28] and Social Cognitive Theory [11] which identify the need for developing supportive environments, raising awareness, developing knowledge and skills, encouraging cognitive processing of the information and reinforcing messages over time. Creating an environment conducive to change is achieved via stakeholder support and involvement, which is an ongoing component of the program, achieved through twice yearly meetings of the practitioner and veteran reference groups, quarterly meetings with the national representative steering committee and regular attendance at health professional meetings. Face-to face meetings are called as required with national bodies for selected topics. Topic selection is always based on an identified medication-related problem, where data analysis has revealed a problem which is amenable to intervention by audit and feedback and which has the support of the clinical reference group and stakeholders. Problems which are not measureable in the data are not implemented under this program. An intervention plan is developed which includes clear objectives, the strategies to meet each objective and process impact and outcome indicators for evaluation. This plan is endorsed by all groups for each intervention. The material includes call to action questions to promote cognitive processing. Stakeholder feedback through our reference groups, call centre, e-mail comments line and one-page response forms identify both barriers and enablers to continue to improve the program. The evaluation results are routinely fed-back to all reference groups and inform the ongoing interventions. Further, the ongoing nature of the program enables repeat messages over time.

While it could be argued that programs such as this should be evaluated using more rigorous methods, such as randomized controlled designs, we advocate that this program represents true implementation science. Previous randomized controlled trial data have demonstrated the efficacy of audit and feedback. Similarly, randomized controlled trial data were available for all Veterans’ MATES interventions targeting medicine efficacy and either randomised controlled trial or well conducted observational evidence is available for all medicine safety issues targeted. For this reason, this program sought to implement the evidence, and as a consequence used health program evaluation methods for the analysis. For this reason, we are limited to methods of evaluation such as time series or non-equivalent groups. As this program was a national program, for the majority of interventions no comparison group was able to be selected, thus time-series was the method used. A further challenge in evaluating ongoing programs are the occurrence of simultaneous interventions, or interventions immediately prior or after which may impact on the evalution and lead to over-estimates or under-estimates of results. This may occur due to changes in co-payments, product withdrawals, changes to subsidy and changes in safety or efficacy or indications. While at times these environmental changes may obscure intervention effects, well targeted programs can take advantage of these opportunities to enhance intervention effect, for example by reinforcing safety messages and giving clear advice on what to use instead. The consistency of our results, in terms of relative effect size, across a range of topics and the consistency of that effect with existing studies using controlled designs [7] suggests our evaluation is likely to be estimating true effect.

We have demonstrated that a well designed program using proven techniques from implementation research, designed with local characteristics in mind and in consultation with key stakeholders can be effective. The Australian medicines and health environment has been exposed to a formal National Medicines Policy for a decade [29]. This policy initiative has funded national quality use of medicines programs, for example the National Prescribing Service, which has been in operation for about 10 years. While this environment may predispose both health practitioners and consumers to be favourable to the types of interventions implemented by the Veterans’ MATES program, the practice changes reported in this paper are over-and-above those that have been achieved through these other policy initiatives [30-32].

## Conclusions

The Veterans’ MATES program has been successful in achieving positive, significant changes in medicines and health service use over time. Educational interventions with a clear, single message tended to be most successful in changing practice as were those which aimed to increase use. The Veterans’ MATES program provides a model that could be replicated in other settings such as a national health service or a health insurance organisation where bridging the evidence-practice gap is proving a significant challenge.

## Competing interests

The authors have no competing interests to declare.

## Authors’ contributions

EER conceived of and designed the study, participated in data analysis and drafted the manuscript. LKE participated in data analysis and drafted the manuscript. ENR and NP performed the data and statistical analyses and assisted in study design and drafting of the manuscript. JB participated in data analysis and critically revised the manuscript for important intellectual content. VL, PR, RP, and GK assisted in study design and critically revised the manuscript for important intellectual content. AG conceived of and designed the study, and critically revised the manuscript for important intellectual content. All authors read and approved the final manuscript.

## Pre-publication history

The pre-publication history for this paper can be accessed here:

http://www.biomedcentral.com/1472-6963/13/514/prepub
